# Equipment-Free Incubation of Recombinase Polymerase Amplification Reactions Using Body Heat

**DOI:** 10.1371/journal.pone.0112146

**Published:** 2014-11-05

**Authors:** Zachary Austin Crannell, Brittany Rohrman, Rebecca Richards-Kortum

**Affiliations:** Department of Bioengineering, Rice University, Houston, Texas, United States of America; Texas A&M University, United States of America

## Abstract

The development of isothermal amplification platforms for nucleic acid detection has the potential to increase access to molecular diagnostics in low resource settings; however, simple, low-cost methods for heating samples are required to perform reactions. In this study, we demonstrated that human body heat may be harnessed to incubate recombinase polymerase amplification (RPA) reactions for isothermal amplification of HIV-1 DNA. After measuring the temperature of mock reactions at 4 body locations, the axilla was chosen as the ideal site for comfortable, convenient incubation. Using commonly available materials, 3 methods for securing RPA reactions to the body were characterized. Finally, RPA reactions were incubated using body heat while control RPA reactions were incubated in a heat block. At room temperature, all reactions with 10 copies of HIV-1 DNA and 90% of reactions with 100 copies of HIV-1 DNA tested positive when incubated with body heat. In a cold room with an ambient temperature of 10 degrees Celsius, all reactions containing 10 copies or 100 copies of HIV-1 DNA tested positive when incubated with body heat. These results suggest that human body heat may provide an extremely low-cost solution for incubating RPA reactions in low resource settings.

## Introduction

Polymerase chain reaction (PCR) is widely considered to be the gold standard for sensitive and specific diagnosis of many infectious diseases. Because PCR amplifies trace levels of DNA to detectable levels, this technique is often orders of magnitude more sensitive than other diagnostic methods such as microscopy or antibody-based assays [1]–[3]. PCR is also highly specific and can be used to differentiate between similar organisms by detection of specific nucleic acid sequences. However, PCR requires access to expensive thermal cycling equipment that is frequently unavailable in low-resource settings where the infectious disease burden is greatest. Even at centralized diagnostic centers in low resource settings, where thermal cyclers and technical expertise are available, PCR may be impractical due to the unavailability of battery powered thermal cyclers or frequent power outages [4].

A number of platforms have been developed to amplify nucleic acids at a single temperature, thus alleviating the need for thermal cycling equipment [5]–[9]. Because isothermal amplification methods require only a single temperature, these platforms can be implemented using a simple, fixed-temperature heater, which costs at least an order of magnitude less than a thermal cycler [10]. In addition to commercially available heaters, a number of research groups have developed battery-powered heaters or exothermal chemical heaters that maintain an appropriate reaction temperature without external power. For example, Myers et al. designed a battery-powered heater and LaBarre et al. coupled an exothermic reaction with an engineered phase change material to enable incubation of loop-mediated amplification (LAMP) reactions at the point of care [11], [12]. The design constraints for such heaters, such as temperature set-point and stability, highly depend on the intended isothermal amplification platform and ambient temperature range.

One such isothermal platform, recombinase polymerase amplification (RPA), offers significant advantages for both instrumentation and assay development. RPA is tolerant to impure samples, amplifies DNA to detectable levels in as few as 5 minutes, and is available in a lyophilized form that can be transported to the point of care without requiring cold chain storage [7], [13], [14]. Lateral flow strips may be used for detection of amplified RPA products in low resource settings. In addition, RPA operates at a wide range of temperatures [7]. TwistDx recommends an incubation temperature of 37 degrees Celsius (the temperature of the human body) but notes that amplification may occur at temperatures as low as 25 degrees Celsius by using additional magnesium acetate, extending incubation time, and agitating reactions later in the incubation period [13]. Others have shown that even without adjusting the biochemistry of reactions, RPA retains reliable functionality between 31 and 43 degrees Celsius [15]. Although the possibility of incubating RPA reactions using body heat has been mentioned in previous work [5], [16], to the best of our knowledge, there are no examples of harnessing body heat to perform RPA in the literature.

In this paper, we explored the feasibility of using body heat to incubate RPA reactions for amplification of HIV-1 DNA. We chose this assay because detection of HIV-1 proviral DNA is an established method for early infant diagnosis [17], and the HIV-1 DNA RPA assay used here has been well-characterized elsewhere [18]. First we measured the temperature of mock reactions incubated at 4 body locations chosen to allow comfortable, convenient incubation. After demonstrating that the axilla is the ideal location for incubation, we investigated 3 commonly available materials to secure RPA reactions to the body. We also studied the effect of ambient conditions on incubation temperature to determine the ambient temperature range for which incubation with body heat may be feasible. Finally, RPA reactions were incubated using body heat while control RPA reactions were incubated in a heat block to demonstrate that body heat may be harnessed to enable isothermal amplification of HIV-1 DNA.

## Methods

### Ethics statement

Ten normal, healthy volunteers were recruited for this study in accordance with protocol 14-211E, approved specifically for this study by the Rice University Internal Review Board. Informed, written consent was given by all volunteers in accordance with the protocol.

### Body temperature measurements

To estimate the temperature that an RPA reaction would reach if incubated using body heat, the temperature of mock RPA reactions was measured at various body locations. A mock reaction consisted of a 2 mL microcentrifuge tube filled with 50 µL of water. The temperature of each mock reaction was monitored via a wire thermocouple probe threaded through a small hole in the top of the tube. The thermocouple probe was attached to a thermocouple measurement device with a USB interface (NI USB – TC01, National Instruments, USA), and thermal measurements were recorded every second. Volunteers held the tube for 45 minutes at 4 body locations chosen to allow comfortable, convenient incubation of RPA reactions. Tubes were held in a closed fist, placed in a rear trouser pocket, held in the axilla (outside of clothing), or taped to the abdomen (under clothing). The temperature of mock reactions at each body location was measured for five volunteers. Because mock reactions were in thermal equilibrium with the ambient temperature before incubation, the initial temperature of each mock reaction was defined to be the ambient temperature for each experiment. To analyze the data for each body location, the average temperature over time was calculated for each volunteer. Then, the mean and standard deviation of those values was computed.

### Evaluation of methods for securing tubes

**Figure 1:**
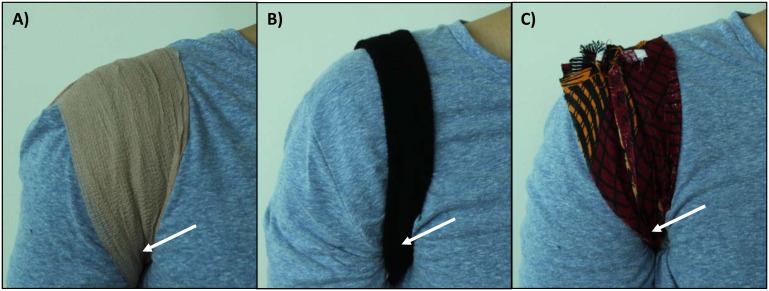
Methods for securing tubes. Mock RPA reactions were secured in the axilla using (A) a bandage, (B) an elastic sweatband, and (C) a strip of African chitenje fabric. An arrow is shown in each panel to indicate the approximate position of the tube, which is covered by material.

To allow convenient incubation of RPA reactions under the arm, several methods were tested for securing tubes to the body. Mock reactions were secured by wrapping a 10 cm wide bandage ($12, CVS Pharmacy, USA), applying a 5 cm wide elastic sweat band ($1, Academy Sports and Outdoors, USA), and tying an 8 cm wide strip of cotton cloth (African chitenje fabric, approximately $4 per yard, outdoor market, Malawi) over the shoulder and under the arm (Figure 1). Volunteers incubated a tube containing 50 µL of water for 45 minutes using each method while the temperature was measured as previously described. These measurements were taken for five volunteers.

### Effect of ambient conditions on incubation temperature

The effect of ambient conditions on incubation temperature was assessed. Variation in ambient conditions was simulated by performing measurements in a cold room, in an air conditioned office, and outside in the Houston summer sun (approximately 4, 10, 22, and 38 degrees Celsius, respectively). Volunteers incubated a microcentrifuge tube containing 50 µL water secured in the axilla with a strip of cotton chitenje fabric for 15 minutes in each environment. Incubations were performed for fifteen minutes to minimize the discomfort to the volunteers when sitting in direct sunlight and in the cold room. Most volunteers in the cold room wore warm clothing over the secured tube. The temperature was measured as previously described in each environment for five volunteers.

### Incubation of RPA reactions using body heat

RPA reactions were assembled according to the manufacturer’s instructions (TwistAmp nfo kit, TwistDx, United Kingdom) using sequences published previously that target and amplify the HIV-1 *pol* gene [18]. Each 50 µL reaction contained 29.5 µL rehydration buffer, 2.1 µL biotin-labeled forward primer (5′-[biotin]-TGGCAGTATTCATTCACAATTTTAAAAGAAAAGG-3′), 2.1 µL reverse primer (5′-CCCGAAAATTTTGAATTTTTGTAATTTGTTTTTG-3′), 0.6 µL FAM-labeled nfo probe (5′-[FAM]-TGCTATTATGTCTACTATTCTTTCCCCTGC[dSpacer]CTGTACCCCCCAATCCCC[C3 Spacer]-3′), 3.2 µL water, one enzyme pellet, 2.5 µL magnesium acetate, and 10 µL of water or HIV-1 DNA template. All DNA oligonucleotides were purchased from Integrated DNA Technologies (Novato, USA). HIV-1 DNA samples contained a background of 10 ng of human genomic DNA and a total of 0, 10, or 100 copies of the plasmid pHIV-IRES-eYFPΔEnvΔVifΔVpr, a generous gift from R. Sutton [19].

RPA reactions were incubated using the body heat of ten volunteers at room temperature in an office or laboratory with an ambient temperature between 21 and 26 degrees Celsius. RPA reactions were also incubated using the same method in a cold room with an ambient temperature of 10 degrees Celsius. Each volunteer incubated three RPA reactions containing 0, 10, or 100 HIV-1 DNA copies in 0.5 mL tubes. This tube size was chosen so that screw-caps could be used, which minimize the formation of aerosols that may lead to amplicon contamination. The 0.5 mL tubes containing RPA reactions were placed in a 5 cm×5 cm zipper locking plastic bag (International Plastics, USA), incubated under the volunteer’s arm, and secured with a strip of cotton chitenje fabric. For each volunteer, three control reactions (also containing 0, 10, or 100 HIV-1 DNA copies) were incubated at 37 degrees Celsius in a VWR heat block (13259-000, VWR, USA). For experiments performed at room temperature and at 10 degrees Celsius, RPA reactions were incubated for 20 minutes and 30 minutes, respectively. Experiments performed in the cold room were incubated for 30 minutes because previous work has shown that an extended incubation time may increase RPA sensitivity at temperatures below 30 degrees Celsius [20]. After incubation, all reactions were placed on ice to halt amplification.

Lateral flow detection of the amplicons dual-labeled with FAM and biotin was accomplished using commercially available lateral flow strips according to the manufacturer’s instructions (MGHD 1, TwistDx, United Kingdom). For each RPA reaction, 2 µL of amplified product was diluted in 98 µL of supplied running buffer. Ten microliters of diluted product was then added to the sample pad of the lateral flow strip, and the strip was placed in a well of a 96-well plate containing an excess of running buffer. After three minutes the strips were removed and scanned with a flatbed scanner. The signal-to-background ratio (SBR) of the detection region on the lateral flow strips was assessed as previously described [21]. The SBR threshold for a positive sample was defined as three standard deviations above the average SBR of all negative samples incubated in the heat block.

## Results

**Figure 2:**
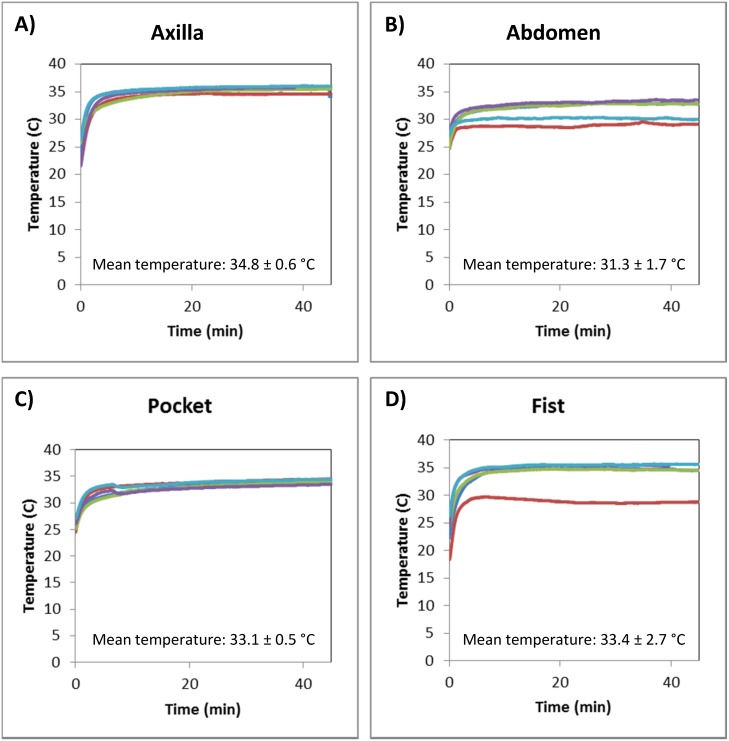
Temperature of mock reactions incubated at different body locations. Each plot shows the temperature traces of mock RPA reactions incubated by 5 volunteers at 1 of 4 body location tested. Mock reactions were (A) held in the axilla, (B) taped to the abdomen, (C) placed in a rear trouser pocket, and (D) held in a closed fist.

The temperature of mock RPA reactions was measured at various body locations to estimate the temperature that an RPA reaction would reach if incubated using body heat. Figure 2 shows the temperature traces of mock RPA reactions incubated by 5 volunteers at 4 body locations. Mock reactions held in the axilla outside of clothing (Fig. 2A), taped to the abdomen under clothing (Fig. 2B), placed in a rear trouser pocket (Fig. 2C), and held in a closed fist (Fig. 2D) had average temperatures of 34.8±0.6, 31.3±1.7, 33.1±0.5, and 33.4±2.7 degrees Celsius, respectively. In less than three minutes, all mock reactions reached a temperature of 31 degrees Celsius, the temperature required for all RPA reactions to amplify DNA to detectable levels [15]. Because the temperature of mock reactions was closest to the temperature recommended for RPA (37 degrees Celsius) when incubated in the axilla, this site was chosen as the site of incubation for all following experiments.

**Figure 3:**
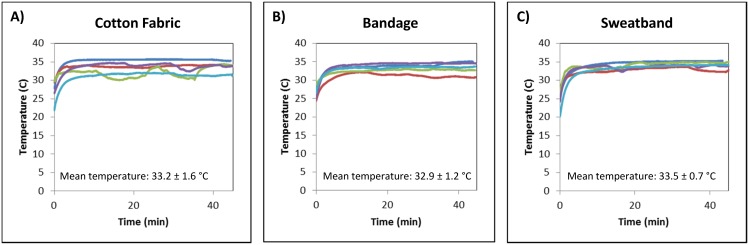
Temperature of mock reactions secured to the body with different materials. Each plot shows the temperature traces of mock RPA reactions incubated by 5 volunteers using 1 of 3 different materials. Materials tested included (A) a strip of cotton fabric, (B) a bandage, and (C) a sweatband.

Several methods were tested for securing tubes to the body to allow convenient incubation of RPA reactions in the axilla. Figure 3 shows the temperature traces of mock RPA reactions incubated by 5 volunteers using 3 different methods. Mock reactions secured with a strip of cotton chitenje fabric (Fig. 3A), a bandage (Fig. 3B), and an elastic sweatband (Fig. 3C) reached an average temperature of 33.2±1.6, 32.9±1.2, and 33.5±0.7 degrees Celsius, respectively. The average time to reach 31 degrees Celsius using the chitenje fabric, a bandage, and an elastic sweatband was 2.0±2.7, 2.5±1.8, and 2.1±1.0 minutes, respectively. Because all methods produced similar temperatures, and cotton fabric is inexpensive and widely available in developing countries, tubes were secured with a strip of cotton fabric for all following experiments.

**Figure 4:**
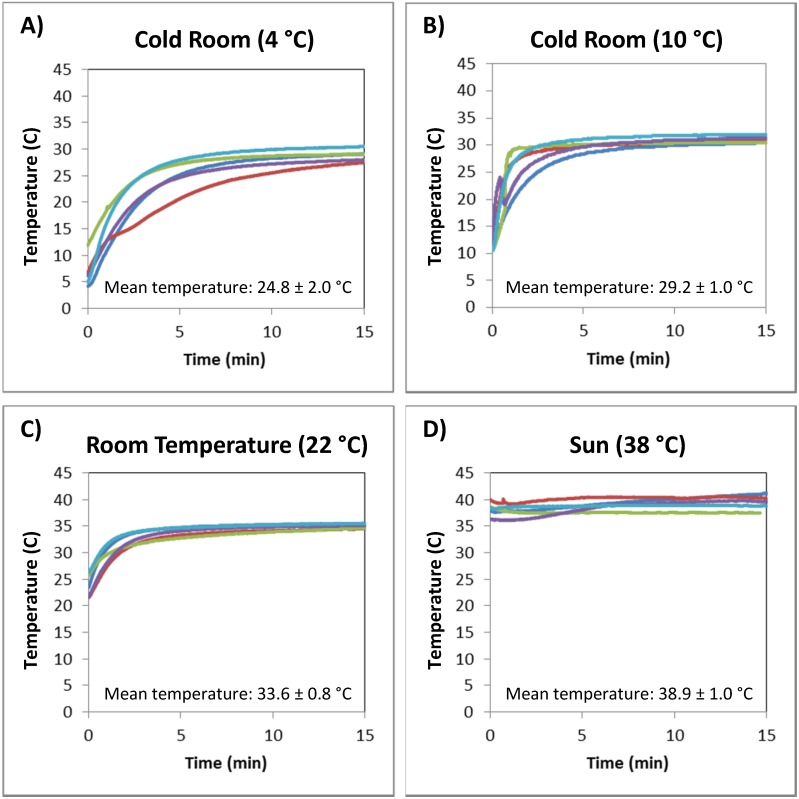
Effect of ambient conditions on mock reaction temperature. Each plot shows temperature traces of mock RPA reactions incubated under the arms of 5 volunteers in 1 of 4 environments with different ambient conditions. Mock reactions were incubated (A) in a cold room at 4 degrees Celsius, (B) in a cold room at 10 degrees Celsius, (C) at room temperature, and (D) in the Houston summer sun.

The effect of ambient conditions on incubation temperature was assessed when tubes were secured in the axilla with cotton fabric. Figure 4 shows temperature traces of mock RPA reactions incubated under the arms of 5 volunteers in four environments with different ambient conditions. Tubes incubated in a cold room at 4 degrees Celsius (Fig. 4A), in a cold room at 10 degrees Celsius (Fig. 4B), at room temperature (Fig. 4C), and in the Houston summer sun (Fig. 4D) reached an average temperature of 24.8±2.0, 29.2±1.0, 33.6±0.8, and 38.9±1.0 degrees Celsius, respectively. These results suggest that this method is feasible for incubation of RPA reactions near room temperature and at higher temperatures, but may not be feasible when the ambient temperature is near freezing.

To demonstrate that body heat may be harnessed to incubate RPA reactions, RPA reactions were secured with a strip of cotton fabric and incubated in the axilla of ten volunteers in an office or laboratory at room temperature and in a cold room at 10 degrees Celsius, while control RPA reactions were incubated in a heat block. For experiments performed at room temperature, all reactions containing no HIV-1 DNA tested negative for both heating methods (Table 1). All reactions containing HIV-1 DNA tested positive when heated in a heat block, while 100% of reactions with 10 copies of HIV-1 DNA and 90% of reactions with 100 copies of HIV-1 DNA tested positive when incubated with body heat. For experiments performed at 10 degrees Celsius, all reactions containing no HIV-1 DNA tested negative for both heating methods (Table 2). When heated in a heat block, all control reactions containing 100 copies of HIV-1 DNA tested positive, and 90% of reactions with 10 copies of HIV-1 DNA tested positive. The false negative sample may have been due to an experimental error. When samples were incubated with body heat, all reactions containing HIV-1 DNA tested positive. For both room temperature and cold room experiments, there was no significant difference between the signal-to-background ratio of the lateral flow strips for control reactions and reactions incubated with body heat when compared using a paired, one-tailed t-test.

**Table 1:** Performance of body heat versus a heat block for incubating RPA reactions at room temperature.

Number ofcopies	Percent positive,Body incubation	Percent positive,Control	Average differencein SBR
0	0% (0/10)	0% (0/10)	0.01 (p = 0.17)
10	100% (10/10)	100% (10/10)	0.08 (p = 0.29)
100	90% (9/10)	100% (10/10)	–0.07 (p = 0.37)

**Table 2:** Performance of body heat versus a heat block for incubating RPA reactions in a cold room.

Number ofcopies	Percent positive,Body incubation	Percent positive,Control	Average differencein SBR
0	0% (0/10)	0% (0/10)	0.00 (p = 0.43)
10	100% (10/10)	90% (9/10)	0.05 (p = 0.35)
100	100% (10/10)	100% (10/10)	0.39 (p = 0.06)

## Discussion

We have demonstrated that RPA reactions may be incubated using body heat for amplification of HIV-1 DNA. Within a certain operating range, the temperature using this method is consistent over time and varies little from person to person. This method may be modified for convenience, as reactions may be incubated at several body locations and secured using available materials that may be reused for many experiments. The use of a plastic bag to contain reactions, while optional, may prevent contamination and provide a protective barrier between the user and the reaction components, which already pose little risk to the user. Temperature profiles and reaction results were not significantly affected by clothing material worn by the volunteer. Incubation of reactions using body heat is extremely inexpensive, as it obviates the need for any heating equipment, and the only consumables required are tubes and pipette tips. The method described here for incubating RPA reactions using body heat may be combined with any suitable DNA extraction method that is compatible with RPA. As RPA is tolerant to sample impurities, simple lysis methods such as boiling may adequately prepare samples for amplification [14]. In addition, this incubation method is compatible with other detection methods, including enclosed systems designed to reduce amplicon contamination, which may be more appropriate for point-of-care settings [22].

There are several disadvantages of using body heat to incubate RPA reactions, but most may be easily mitigated. One drawback of the axilla as a site for incubation is the slight physical discomfort associated with the presence of the tubes. In addition, incubating tubes under one’s arm may seem unhygienic or strange to the user. To address these issues, additional padding or material may be added to increase comfort. When securing tubes under the arm using a strip of cotton fabric, there is a small risk that the tubes may become dislodged. This problem may be solved by sewing a pocket or pouch on the cotton strip to provide a holder for the tubes. Another potential disadvantage of this method is that incubation with body heat may not be feasible in colder climates when the temperature is below 10 degrees Celsius.

Finally, the sensitivity of DNA amplification was slightly lower when reactions were incubated with body heat at room temperature, as only 9 of 10 reactions with 100 copies of HIV-1 DNA tested positive. Notably, the reaction containing 100 copies of HIV-1 DNA that was classified as negative produced a faintly visible line at the test zone of the lateral flow strip (Fig. S1B); however, the SBR was slightly lower than the threshold for positive samples. To ensure that DNA is amplified to detectable levels and that results are clearly positive on lateral flow strips (Fig. S1), a longer incubation time may increase the SBR of lateral flow strip results, thereby increasing assay sensitivity. Once the incubation time is optimized, this method may serve as a low-cost, simple method for incubating RPA reactions in low resource settings.

## Supporting Information

Figure S1 **Signal-to-background ratios of lateral flow strips.** The SBRs for four representative lateral flow strips are given to the right of the raw images for each strip: (A) a negative strip, (B) a false negative strip, in which a faint line is visible but the SBR falls just below the threshold for positive strips, (C) a weakly positive strip, and (D) a strongly positive strip.
(TIFF)
