# Supplementary figures and images for: Equipment-Free Incubation of Recombinase Polymerase Amplification Reactions Using Body Heat
Source: PLoS One. 2014 Nov 5;9(11):e112146. doi: 10.1371/journal.pone.0112146 (PMC4221156; doi:10.1371/journal.pone.0112146)

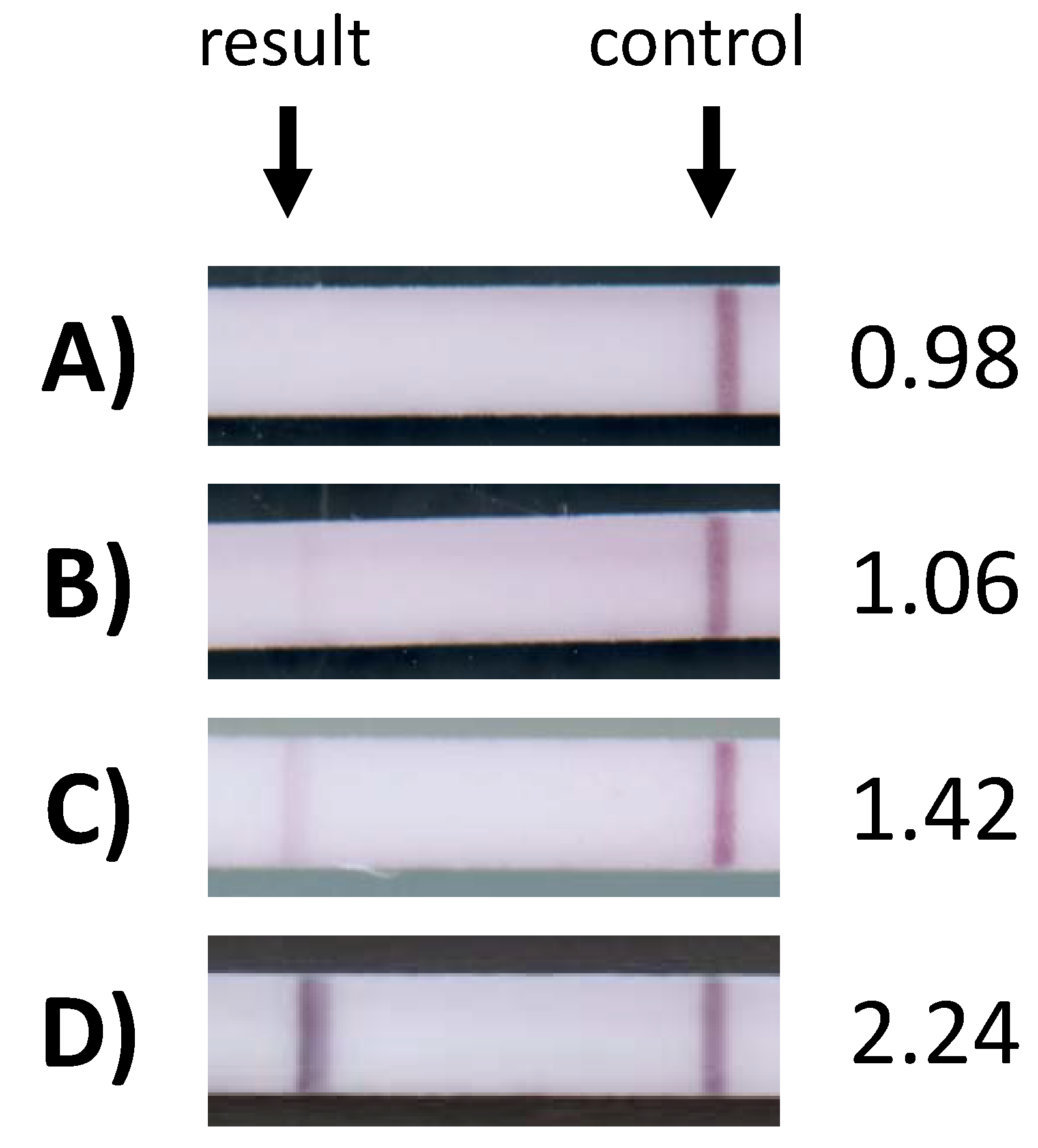

Supplement: Figure S1 — Signal-to-background ratios of lateral flow strips. The SBRs for four representative lateral flow strips are given to the right of the raw images for each strip: (A) a negative strip, (B) a false negative strip, in which a faint line is visible but the SBR falls just below the threshold for positive strips, (C) a weakly positive strip, and (D) a strongly positive strip. (TIFF) [file pone.0112146.s001.tiff]
